# Antimicrobial drug pricing

**DOI:** 10.1038/s43856-024-00594-9

**Published:** 2024-10-11

**Authors:** Avaneesh Kumar Pandey, Nusrat Shafiq, Ashish Kumar Kakkar, Samir Malhotra, Beth Woods, Christopher Little, Tom Rhodes, Harriet Tuson, Zeshan Riaz, Tom Ashfield, Michael Corley, Ioannis Baltas

**Affiliations:** 1 Department of Pharmacology, Postgraduate Institute of Medical Education and Research, Chandigarh, India; 2 Centre for Health Economics, University of York, York, UK; 3 Pfizer UK, Tadworth, UK; 4 British Society for Antimicrobial Chemotherapy, London, UK; 5 Infection, Immunity & Inflammation Department, University College London Institute of Child Health, London, UK; 6 Department of Clinical Microbiology, University College London Hospitals NHS Foundation Trust, London, UK

**Keywords:** Antimicrobial resistance, Drug development

## Abstract

Despite the constant development of antimicrobial resistance (AMR), few new antimicrobials are currently becoming available clinically. Alternative approaches, such as different mechanisms to fund their use, are being explored to encourage development of new antimicrobials.

Antimicrobial resistance (AMR) occurs when microbes develop resistance to the drugs that are being used to eliminate them. AMR is a global health emergency that necessitates constant development of new antimicrobial drugs. However, in recent years few new antimicrobial drugs have become available to clinicians. One factor impacting the antimicrobial drug development pipeline is using the traditional approach to pricing of drugs, in which payment is made based on the volume purchased. In this Viewpoint experts with interests in antimicrobial drug development discuss why this situation has arisen and how alternative pricing strategies could encourage the development of new antimicrobials and further optimise their use.

## Avaneesh Kumar Pandey, Nusrat Shafiq, Ashish Kumar Kakkar & Samir Malhotra

**Figure:**
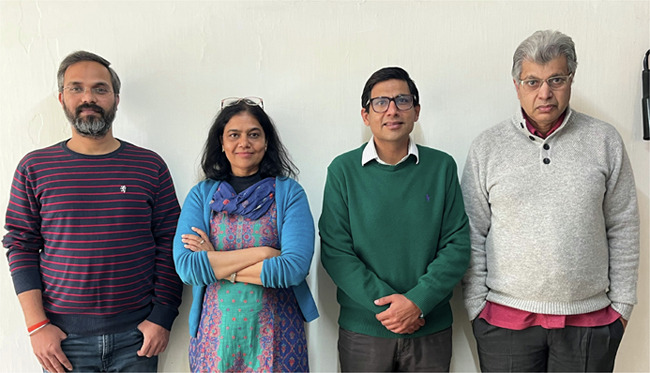
(Left to right) Avaneesh Kumar Pandey, Nusrat Shafiq, Ashish Kumar Kakkar, Samir Malhotra Seema Prajapati

We work as pharmacologists (AKP) and clinical pharmacologists (NS, AKK and SM) in a tertiary level healthcare, medical education and research centre in North India. Our centre is specifically focused on clinical pharmacological research in antimicrobials and optimum antimicrobial stewardship. Drug pricing is of particular interest to us as we aim to further the implementation of antimicrobial stewardship in resource limited settings.

Appropriate usage and pricing of antimicrobials is an ongoing issue, as AMR is projected to result in economic costs ranging from a shortfall of 1.1–3.8% in global Gross Domestic Product (GDP) annually by the year 2050 with low-income countries expected to be affected the most^1^. The World Health Organization (WHO) essential medicines list (2023) comprises medicines that the WHO views as required for a health system to function adequately. This list includes 250 antimicrobials, and the WHO highlights that it is important that medicines of acceptable quality standards are always available in functioning health systems^2^. This is only possible if they are at an affordable price in the communities where they are needed.

When drugs are initially developed, the inventor can control pricing by patenting the drug. Patents prevent the copying, manufacturing or selling of an invention by people other than the inventor without their permission for a period of time after disclosure of the invention. Once drugs are off patent, other manufacturers can produce and sell these drugs, and they are called generics^3^. The original manufacturers can also provide compulsory or voluntary licenses that allow manufacturers to make generics in countries where they are not marketing the product. This can make the drugs both available and affordable^4^. Generics are often 80–85% cheaper than branded medicines^5^. However, low cost, good quality generics are often not sustainably available in many economies across the globe^6^. For example, a survey of 65 generic clarithromycin products manufactured in different countries for sale in the USA, failed to achieve various quality parameters specified by the regulators when compared with the drug formulations developed and produced by the original company that developed and produced the product^7^. Additionally, when there are sudden surges in prices of antimicrobials, as occurred for pyrimethamine (Daraprim) in the USA, medical management can become unsustainable^8,9^.

Antimicrobials are unique among pharmaceutical agents in many ways. There is a paradox that if you use a particular antibiotic more, the chances of selection of resistant pathogens increase, potentially making the particular antimicrobial less effective. With better living conditions, access to water, sanitation and hygiene and vaccine coverage, bacterial infections are less frequent and so are seen as less of a healthcare priority. These factors make investment in antimicrobials a less lucrative scenario for pharmaceutical companies. Moreover, many clinical scenarios require only a short course of antibiotics, such as the treatment of community-acquired pneumonia and urinary tract infection, amongst others.

Antibiotic drug pricing and regulation across the world vary significantly due to differences in healthcare systems, government policies, economic factors, and international agreements. Various systems in place for regulating drug prices include price controls, reference pricing, and health technology assessments to assess the cost-effectiveness of antimicrobials. Government intervention ranges from none, where prices are largely determined by market forces, to significant involvement in setting drug prices through negotiations and regulatory frameworks. In the United States, the market or manufacturers typically have authority to set the prices for their products. On the other hand, in several European nations, such as Germany and Sweden, there are regulations that impact drug pricing, although manufacturers still retain some flexibility in their ability to set prices^10^. In India, the government regulates the prices of certain drugs, while the prices of others can be set by the manufacturers. The Drug Price Control Order (DPCO) regulates the prices of essential medicines by setting price ceilings. This ensures that these essential medications remain affordable and accessible to all segments of society. The DPCO aims to strike a balance between the interests of consumers and pharmaceutical companies by keeping drug prices in check while ensuring a steady supply of essential medicines^11^. Since the issues affecting drug pricing are multifactorial, effective solutions need to have a multi-pronged approach too. Collaboration between governments and international organisations can promote price transparency and reduce overall cost through negotiations, pooled procurement systems^12^, and voluntary licensing agreements^13^. Pooled procurement, where multiple entities combine their demand for antimicrobials to negotiate better prices and voluntarily licensing agreements that allows other manufacturers to produce and sell the patented agents under specific terms and conditions can ensure affordable pricing for essential antibiotics without compromising on their quality.

Additionally, the adoption of differential pricing structures by pharmaceutical companies can consider an individual country or region’s ability to pay^14^. It is essential to strike a balance between incentivizing innovation and safeguarding public health by ensuring equitable access to antimicrobial treatments. DPCO could play an important role by taking both perspectives into consideration^11^. Public-private partnerships and increased funding for research initiatives can help stimulate innovation while prioritising global health needs. Public-private partnerships that decentralise pharmaceutical manufacturing could promote competitive pricing and ensure wider availability, especially of traditional antimicrobials such as penicillins. This would require rejuvenation and strengthening of quality control systems and manufacturing capacity where it is not already present. International consideration of the need to strengthen quality control systems for generic manufacturing units is also important, as this enables best practices to be shared globally. Antimicrobials of suboptimal quality pose several serious issues ranging from ineffectiveness, antimicrobial resistance, toxicity, economic wastage to loss of public confidence. Strategies must also consider antimicrobial stewardship, to ensure existing antibiotics are used optimally and so are efficacious for as long as possible. As we conclude, we are keen to emphasise that antimicrobial pricing should be a matter of global concern. Inputs from several key stakeholders, such as those listed above, are required to develop policies to address this critical issue urgently.

## Beth Woods

**Figure:**
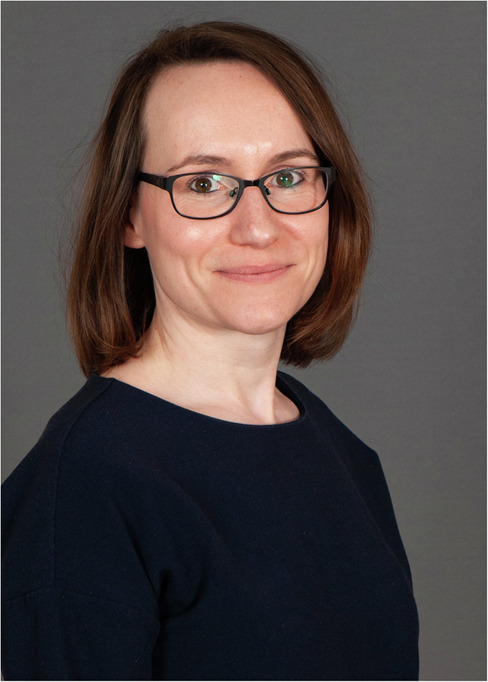
the Author

I am a Senior Research Fellow at the Centre for Health Economics, University of York. I have worked on frameworks for the value assessment of new antibiotics, and on the value assessments that informed the recent UK subscription payment model for cefiderocol and ceftazidime with avibactam, two antibiotic combinations that have recently become available in England.

Because there are few new antimicrobials that are effective against resistant pathogens, policymakers are seeking novel ways to incentivise their development and commercialisation. Initiatives in the USA and UK are exploring the potential for payment models that provide a fixed payment level based on overall product value, and which is not influenced by the volume of drug sales. These subscription payment models aim to support antimicrobial stewardship goals and provide more consistent revenues over time. This is important as valuable antimicrobials may only be used in significant volumes in the future when drug-resistant infections become more common. Under conventional payment models where payment is linked to volume this may result in short-term revenues that are too low to encourage investment in research and development (R&D).

A key question when designing payment models is how much we should pay for a new antimicrobial. Some commentators have focused on ensuring payment is sufficient to cover R&D costs^15^ whereas others have focused on tying payment to value^16^. The problem with an R&D cost-based approach is that it incentivises more R&D spending but not necessarily the development of drugs that offer health or other benefits. A value-based approach offers the potential to signal clearly and consistently what is of value, which should, in theory, help to align drug R&D with the goals of health systems and the needs of current and future patients. A well-designed value-based system can promote population health by balancing incentives for drug development with the impact of drug spending on other health care priorities.

One challenge with implementing value-based subscription payments in this context is that the value of antimicrobials is difficult to estimate. Work undertaken by the Universities of York and Sheffield attempted to assess the health benefits and healthcare cost impacts of two new antimicrobial treatment combinations, cefiderocol and ceftazidime with avibactam, to support the development of National Institute of Health and Care Excellence (NICE) guidance and subscription payment plans for these products. NICE provides evidence-based guidance to health care professionals, whilst ensuring the recommended treatments provide value for money. Our research involved extensive reviews of the literature and mathematical modelling to predict the health and cost impacts of introducing the new products. This revealed that there was limited data available about the number of patients who would be eligible to receive the products today and in the future, and their likely health benefits. Improved linked clinical, prescribing and laboratory data should offer the opportunity to better understand highly resistant infections, and therefore the value of new products. I hope that the UK experience can provide guidance to other health systems about how to move forward with value-based payments for new antimicrobials.

## Christopher Little, Tom Rhodes, Harriet Tuson & Zeshan Riaz

**Figure:**
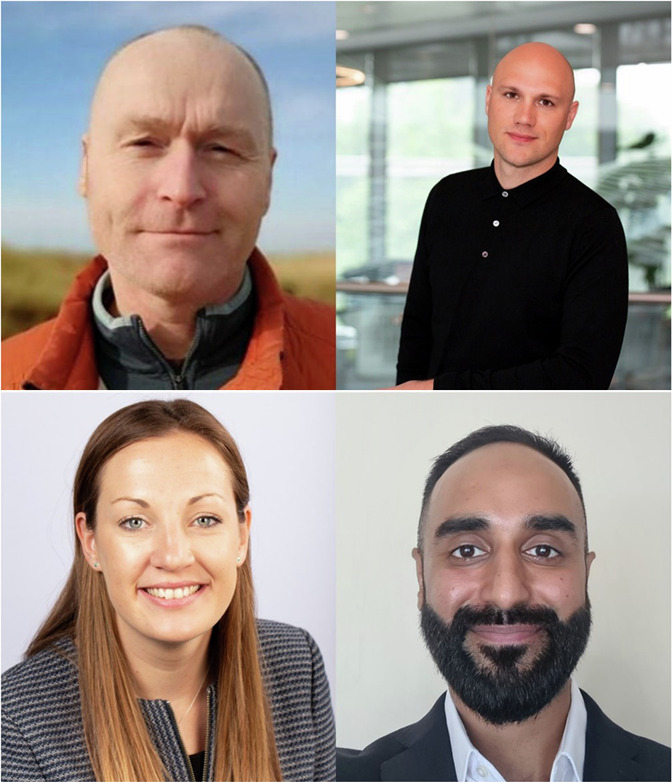
Christopher Little (top left), Tom Rhodes (top right), Harriet Tuson (bottom left) and Zeshan Riaz (bottom right) the Authors

This section of this Viewpoint article has been developed by Pfizer. We work within Pfizer UK on our anti-infectives portfolio within a cross-functional Pfizer team that works across Market Access, Policy and Medical Affairs. We have been engaging with NHS England (NHSE), NICE and as a part of the Association of the British Pharmaceutical Industry (ABPI) AMR forum on the proposals for a permanent subscription model for appraising anti-infectives for use in the UK.

Antimicrobials are a cornerstone of modern medicine, stopping infections from minor injuries becoming life threatening, enabling procedures that would otherwise have high rates of mortality, and limiting spread of infectious diseases through populations. However, due to the rapid evolution of microbes, there is an inevitable emergence of AMR as these treatments are used. To reduce AMR, health care systems have implemented tight restrictions on new medicines, which in turn limits financial returns and disincentivises development. There has been a paucity of research and development into new antimicrobials due to this market failure. This is illustrated by data from The Pew Charitable Trust estimating that in 2020 there were only 43 new antimicrobials in clinical development^17^ in comparison with 4720 immuno-oncology agents^18^. Many large pharmaceutical companies have divested their antimicrobial R&D portfolios, and several innovative biotechnology companies have filed for bankruptcy, despite developing promising new agents^19–21^. As far as we are aware, Pfizer remains one of few large pharmaceutical companies still active in this area.

Despite an uncertain global landscape, it is an exciting time in the UK to be working on anti-infectives and AMR advocacy, with the UK leading the way in exploring new solutions to tackle market failings. In July 2022, NHSE and NICE launched a pilot project to test an innovative approach whereby companies are paid a fixed annual fee for antimicrobials, based on an appraisal of their value to the NHS, instead of the volumes used (i.e., subscription-based contracts). Two anti-infectives were piloted and subscription style contracts were awarded by NHSE. These contracts were the first of their kind globally.

Following the pilot, NHSE has now confirmed its intention to roll out the model more broadly, using simplified criteria and a point-based scoring system^22^. All manufacturers of branded anti-infectives will have the opportunity to apply for consideration within a new permanent model this year. This is an important step in the right direction and will help ensure longer-term strategic investment in anti-infectives.

It will be vital for other developed markets to follow suit in incentivising investment to ensure that the global threat of antimicrobial resistance is tackled head on. Monetary awards should be designed to represent a “fair share” of the global “pull” incentive needed to encourage innovation^23^. There have been some notable developments: an expert panel has proposed a subscription-based model in Canada^24^; Japan is planning a ‘revenue guarantee’ model to ensure access to priority antimicrobials, and the US PASTEUR ACT remains under consideration, which would provide delinked annual revenues to companies in a similar way to the NICE/NHSE model^23^.

## Tom Ashfield & Michael Corley

**Figure:**
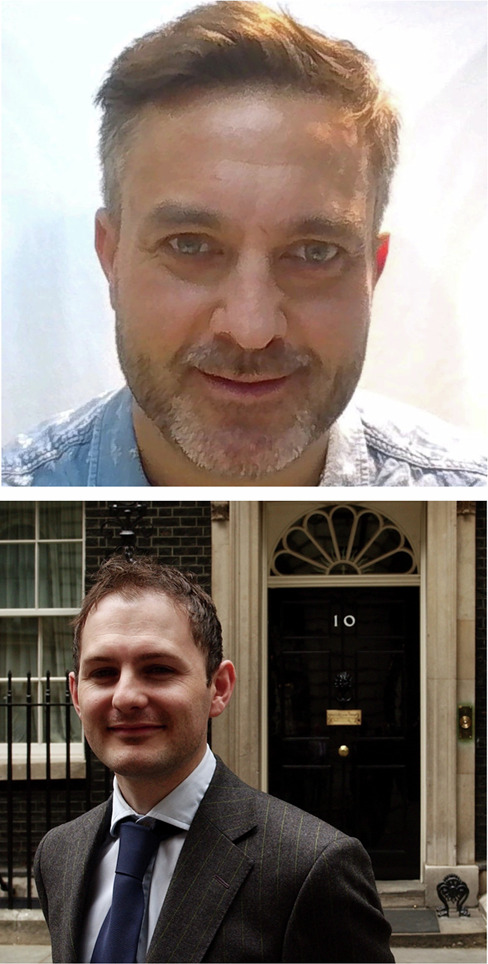
Tom Ashfield (top) and Michael Corley (bottom) the Author

We are representatives of the British Society for Antimicrobial Chemotherapy (BSAC), a global educator, aiming to join the dots between scientific researchers, medical communities and the wider public to stop the growing threat of drug-resistant infections. TA is an elected member of BSAC council, a healthcare strategist and a clinician with expertise in AMR policy reform. TA guided the successful application of a novel antibacterial, active against severe drug resistant infections, within the world’s first volume de-linked payment model. MC is Deputy Chief Executive Officer of BSAC and a senior newspaper journalist. MC provides secretariat to the UK’s All-Party Parliamentary Group on Antimicrobial Resistance, sits on the Steering Committee of the Trinity Challenge, and is a Trustee of the Charades Theatre Company – the charity responsible for producing The Mould that Changed the World (https://www.mouldthatchangedtheworld.com/).

Antimicrobials can be regarded as the fire extinguishers of healthcare^25^. In addition to being used to treat infectious disease, these medicines enable surgery, chemotherapy and invasive procedures to be carried out safely. They can also limit disease outbreaks, avert pandemics, and, with appropriate use, impede the development of AMR^26^. However, the immense value of having efficacious antimicrobials as a part of societal infrastructure is easily overlooked and can only be realised through holistic appraisal.

Traditional pharmaceutical pricing models estimate the value of a single product for treatment of a single disease e.g. blood pressure. This significantly underestimates the value of antimicrobials, in which pathogen-oriented data should be used and the impact on syndromes across a continuum ranging from early infection in community (e.g. urinary tract infection) to life-threatening hospital conditions (e.g. fulminant sepsis requiring organ support) must be appraised.

An integral element, not to be overlooked is enablement value. Antimicrobials enable high-cost invasive procedures such as surgery, transplant and chemotherapy to be undertaken. However, to minimise AMR development this benefit can only be maintained through long-term rationalised and stewarded use of each antibiotic (AMS). It is imperative that any valuation model therefore has AMS at its core and incentivises responsible and appropriate use. An ideal approach allows the value, and pricing to be established for a bundle of solutions that encompass diagnostic technologies in addition to therapeutics.

The holistic value of an antimicrobial extends beyond the individual patient. Significant global disparities exist in access to the most useful of these medicines and there are few new agents in development. The societal value of antimicrobials can be appreciated by understanding how access and supply of them is essential for pandemic preparedness. AMR is the archetypal One Health conundrum (characterised by the World Health Organization as “an integrated, unifying approach to policy that aims to sustainably balance and optimise the health of people, animals and ecosystems”), in which a collaborative transdisciplinary approach is required to optimise health outcomes, for example, through appropriate management of infections we can assure global food security.

AMR needs to be addressed urgently but unfortunately continues to evolve at pace. Meanwhile, reforms to encourage development of antimicrobials are emerging across the globe. One example is the NHSE and NICE subscription model^27^ which provides a framework to encourage development of new drugs through a pull-incentive. In this pricing model, money is provided to developers according to the value that each drug provides, rather than the volume that is used. This approach is an example of value-based economics (VBE) in action, in which equitable resource provision and reimbursement is promoted by focusing on outcomes rather than volumes consumed. Sweden, as a long-term advocate of VBE has successfully embedded this approach within other therapy areas^28^ and is now progressing towards its own pull incentive model for antimicrobials^29^.

In parallel to these innovative payment approaches, it is vital to monitor outcomes and undertake surveillance of product use. In the UK this is being enabled by the UK Antimicrobial Registry (UKAR)^30^, a collaborative effort that sees BSAC work with academic and industry partners to monitor outcomes for patients and to curtail the threat of AMR. Initially, information is being collected on the use of the novel antibiotics that have been accepted into the NHSE/NICE model.

Useful AMR data exists in abundance across other platforms globally, for example Vivli AMR^31^, GLASS (Global Antimicrobial Resistance and Use Surveillance System)^32^ and the ECDC (European Centre for Disease Prevention and Control)^33^. The power of such data can be realised by mapping and linking this vast landscape and then applying it with strategic impact to geography, pathogens and to particular antimicrobials or classes. The Surveillance and Epidemiology of Drug-resistant Infections Consortium (SEDRIC) is a think tank tasked with bridging such gaps (sedric.org.uk).

AMR hits hardest in low- and middle-income countries (LMICs), where its effects are aggravated by mounting inequity linked to gender, race, age, and socioeconomic status. Consequently, AMR poses a significant threat to many of the UN’s 17 Sustainable Development Goals. High-income countries (HICs) must develop a policy of enlightened self-interest by investing in resilient health systems in LMICs. Alarming rates of resistance linked to the deep impact of globalisation will mean that failure to do so will significantly compromise all attempts to protect HICs (including current investments to develop new drugs). When it comes to AMR, no one is safe until everyone is safe.

## Ioannis Baltas

**Figure:**
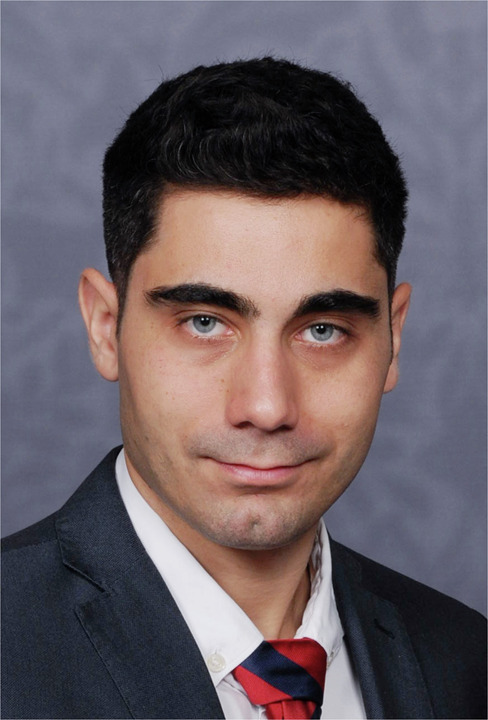
the Author

I am an Infectious Diseases and Medical Microbiology doctor in London, United Kingdom. Having observed first-hand the burden of AMR in my home country, Greece, I am now researching innovative solutions to mitigate AMR and promote AMS. At the launch of the NHSE funding model for antimicrobials, I led a nationwide survey of infectious diseases and medical microbiology consultants, aiming to capture their views about the impact of delinked funding models for antimicrobials on AMR and AMS.

For most drugs, treated patients are the only individuals affected by them, both in terms of therapeutic benefit and side effects. On the contrary, antimicrobials constitute precious societal drugs, offering benefits reaching far beyond the treatment of an individual patient. These benefits are increasingly recognised and grouped using the abbreviation STEDI values (spectrum, transmission, enablement, diversity, insurance)^34^. These values highlight different unique aspects of antimicrobials. They enable other forms of healthcare to happen safely (e.g. surgery, chemotherapy), thus safeguarding investments and innovation in these areas (enablement value). They provide insurance that effective treatments will be available, even if levels of resistance increase (insurance value). Each new antimicrobial protects all others, increasing the number of treatment options available and reducing selection pressure against each individual agent (diversity value). Additionally, prompt effective treatment of resistant infections can reduce transmission to other patients (transmission value), minimising the societal impact of AMR. In order to achieve these benefits, antimicrobials need to be targeted only to resistant bugs and preserve the microbiome (spectrum value). Ultimately, antimicrobials are a finite resource - as a society, we have a limited number of courses of antimicrobials that we can prescribe before rendering them ineffective. This is not observed with other drugs, such as anti-cancer drugs or antihypertensives. In my opinion, antimicrobials should be viewed as public infrastructure that we all need to preserve. Therefore, together with AMS interventions, a constant supply of new antimicrobials is needed to allow ongoing effective treatment of infections. Despite this threat, in 2020, only 41 new antimicrobials were in phase 1 to 3 clinical trials, and they can be viewed by the pharmaceutical industry as commercially unattractive, given the return on investment is typically low due to the low number of doses sold during the time of market exclusivity^35^.

Over the last decade, there has been significant global political push towards trialling innovative funding models for antimicrobials, including delinked models, to address the empty antimicrobial research and development pipeline^35^. In the model set up by England, a set payment is given to a company that enables the NHS to have a right to secured access to a particular antimicrobial. One of the key lessons learned so far from implementing this model is that it is challenging to estimate and renumerate the STEDI values of included antimicrobials, which can far outweigh the financial benefits of treating individual patients^26^. This was particularly pertinent for the insurance, diversity and enablement components of the STEDI framework^36^. There is a significant knowledge gap as to how treatment of an individual patient with antimicrobials affects the wider community and which clinical and policy interventions can optimise the benefits seen. Additionally, lack of real-time epidemiological monitoring of drug-resistant infections in many countries, makes estimating the cost of resistant infections challenging. Performing the necessary studies to assess the impact of STEDI is complex, with significant ethical and financial considerations. Yet, if delinked funding models are to become the primary route for new antimicrobials to enter the market in many countries, standardised research methodology for assessing the values needs to be developed, so that the antimicrobials with the highest societal impact can be selected and reimbursed. This will include drugs with high barrier to resistance, active against the WHO priority pathogens, with data on the impact on transmission and the effect on the microbiome.

A second consideration around delinked funding models is with regards to their effect on everyday clinical practice. Ideally, clinicians should not think at first of cost, but prescribe the best available treatment for patients. Yet we showed that antimicrobial cost strongly influences the decision-making process of a significant percentage of physicians in the UK^36^. If use is delinked from price for new antimicrobials, they might be favoured over equally effective older treatments. However, using new antimicrobials without justification, is generally not favoured by UK clinicians^36^. Reassuringly, both clinician-reported and actual use of ceftazidime-avibactam (a first-line antimicrobial used to treat multi-drug resistance) remained the same post model implementation in England. This suggests that, with appropriate AMS programmes in place, delinked funding models will not lead to overconsumption of last-line treatment options and thus might provide one solution to address the market failure for antimicrobials.
